# Television Viewing Time, Physical Activity, and Mortality Among African Americans

**DOI:** 10.5888/pcd15.170247

**Published:** 2018-01-18

**Authors:** Tasnim F. Imran, Mark Ommerborn, Cheryl Clark, Adolfo Correa, Patricia Dubbert, J. Michael Gaziano, Luc Djoussé

**Affiliations:** 1 Department of Medicine, Brigham and Women’s Hospital, Veterans Affairs Boston Healthcare System, Harvard Medical School Boston, Massachusetts; 2 Department of Medicine, Cardiology Section, Boston Medical Center, Boston University School of Medicine, Boston, Massachusetts; 3 Department of Medicine, Jackson Heart Study, University of Mississippi Medical Center, Jackson, Mississippi; 4 Little Rock Geriatric Research, Education, and Clinical Center and South Central Veterans Affairs Research, Education, and Clinical Center, University of Arkansas for Medical Sciences, Fayetteville, Arkansas

## Abstract

**Background:**

Prolonged television viewing time, a marker of sedentary activity, is independently associated with increased all-cause mortality; however, this association has rarely been studied in African Americans. The objective of our study was to examine the association between television viewing time and mortality among African Americans by using data from the Jackson Heart Study (JHS).

**Methods:**

We studied 5,289 participants from the JHS study who reported television viewing time (h/day) in the JHS baseline questionnaire from 2000 through 2004. Using multivariable Cox regression models adjusted for age, sex, smoking, alcohol use, physical activity, nutrition, prevalent coronary heart disease, chronic kidney disease, diabetes, and hypertension, we computed hazard ratios to examine the association between television viewing time (≤2 h/day, 2–4 h/day, and ≥4 h/day) and mortality.

**Results:**

Participants had a mean age of 55 years, and 64% were women. After a median follow-up of 9.9 years (interquartile range, 9.0–10.7), 615 deaths occurred (data analysis conducted in 2017). Hazard ratios for mortality were 1.08 (0.86–1.37) for television time of 2 to 4 hours per day and 1.48 (95% CI: 1.19–1.83) for television time of greater than or equal to 4 hours per day when compared with those who watched television less than 2 hours per day (*P* trend = .002). When we restricted analyses to those who performed leisure-time activities, the hazard ratios for mortality were 1.10 (95% CI, 0.84–1.45) for television viewing of 2 to 4 hours per day and 1.45 (95% CI, 1.13–1.86) for more than 4 hours per day compared with the less than 2 hours per day.

**Conclusion:**

Our findings suggest that greater television viewing time, even among those who perform leisure-time physical activities, is associated with increased all-cause mortality among African Americans. Thus, it may serve as an indicator of a sedentary lifestyle with potential for intervention.

## Introduction

Television viewing time, as a marker of sitting time, is one of the most prevalent sedentary behaviors in the United States. An estimated 50% to 70% of Americans spend 6 or more hours sitting in a day. In addition to these hours, 20% to 35% spend 4 hours or more daily watching television (1). Recent evidence suggests that greater television viewing time and daily sitting time may be independently associated with adverse health outcomes, such as higher prevalence of diabetes, cardiovascular disease, and all-cause mortality, even after adjusting for physical activity and cardiovascular risk factors (2–6). Although several studies reported the association between physical activity and mortality, the independent effects of different types of sedentary behaviors have not been fully described.

Most studies of the association between physical activity and mortality included white participants only. However, patterns of physical activity and sedentary time may vary across racial/ethnic groups (6). For instance, in the REGARDS (REasons for Geographic and Racial Differences in Stroke) study, which included African Americans from the stroke belt region, found that African Americans had a 6% higher odds of prolonged sedentary time than whites (7). To our knowledge, the association between television viewing time and all-cause mortality among African Americans has not been examined. For example, African Americans are more likely to develop diabetes than non-Hispanic whites and have higher rates of obesity than other racial/ethnic groups (8). If daily television viewing time is associated with increased mortality and morbidity (illness and disease) in this group independent of chronic conditions or level of physical activity, targeted public health interventions to address this issue could significantly affect quality of life and longevity. The objective of our study was to examine the association between television viewing time and mortality among African American participants by using data from the Jackson Heart Study (JHS).

## Methods

JHS is a longitudinal, population-based cohort study that began in 2000 of adults (n = 5,301) living in Hinds, Rankin, and Madison counties in Jackson, Mississippi, who self-identified as African American. The cohort includes men and women aged 21 to 94 years recruited from 4 sources: 31% were prior Jackson area participants in the Atherosclerosis Risk in Communities (ARIC) study; 17% were randomly recruited from the aforementioned counties in Jackson, Mississippi; 30% were volunteers who were identified as representative of the African American population of Jackson on the basis of their demographic characteristics; and 22% were family members of JHS participants who were included for genetic studies. The JHS study design and recruitment process have been described previously (9–11). JHS participants completed a baseline clinical examination and standardized questionnaires from 2000 through 2004. Race/ethnicity was self-reported and confirmed by using the ARIC Household Enumeration Form. Participants were eligible for inclusion in our analysis if they completed a JHS baseline questionnaire and answered the questions about television viewing time. Twelve participants had missing data on time spent on television watching and were therefore excluded from our analysis, yielding a final cohort of 5,289 participants. The JHS study was approved by the University of Mississippi Medical Center Institutional Review Board. All participants gave written informed consent to participate in the study.

Television-viewing time was self-reported and was assessed from JHS standardized baseline questionnaires. Participants were asked how often they watched television: less than 2 hours per day, 2 to 4 hours per day, or more than 4 hours per day. In addition, participants were asked about leisure time physical activities with questions to assess duration, frequency, consistency, and intensity of physical activity in a validated questionnaire, described below (12,13).

The outcome was all-cause mortality, which was assessed in JHS by using death records from the Mississippi Department of Health and the National Death Index. Covariates were demographic variables such as age (years) and sex (male/female) and behavioral risk factors such as cigarette smoking, leisure time physical activity, and alcohol consumption. The baseline survey categorized cigarette smoking as never smoker, former smoker or current smoker and asked participants “Have you smoked more than 400 cigarettes in your lifetime?” and “Do you now smoke cigarettes?” to define current smoking. Alcohol consumption was estimated from a food frequency questionnaire assessing serving sizes of beer, wine, and liquor and frequency of consumption (14). Physical activity was divided into groups in the JHS questionnaires according to the American Heart Association (AHA) categories for leisure time physical activity for adults: 1) poor, no leisure-time physical activity; 2) intermediate, 1 to 149 minutes per week of moderate physical activity or 1 to 74 minutes per week of vigorous physical activity or 1 to 149 minutes per week of moderate and vigorous physical activity; 3) ideal, 150 minutes per week or more of moderate physical activity or 75 minutes or more per week of vigorous physical activity or 150 minutes per week of moderate and physical vigorous activity (15). Physical activity was assessed by the JHS physical activity survey, administered by interview. This survey included information on active living, work, home and garden, and sport and exercise indexes (12). Questionnaire responses were then validated in a subgroup of volunteer participants by using 24-hour accelerometer and pedometer monitoring (13). Nutrition was defined according to AHA nutrition categories, which are based on food components from the Dietary Approaches to Stop Hypertension (DASH) eating pattern: (consuming ≥4.5 cups/d of fruits and vegetables, consuming ≥2 servings/wk of fish, consuming ≥3 servings/d of whole grains, consuming no more than 36 oz/wk of sugar-sweetened beverages, and consuming no more than 1,500 mg/d of sodium). The AHA nutrition categories were 1) poor, adhering to 0–1 components; 2) intermediate, adhering to 2–3 components; and 3) ideal, adhering to 4–5 components (15).

Body mass index (BMI) was calculated as weight in kilograms divided by height in meters squared. Participants were asked about education level in the baseline questionnaire. We categorized education as less than a high school diploma or a high school diploma or higher education. Total cholesterol and high-density lipoprotein (HDL) cholesterol (mg/dL), defined as continuous variables, were obtained from baseline assays that used standard techniques. Hypertension was defined as having a systolic blood pressure greater than or equal to 140 mm Hg, a diastolic blood pressure greater than or equal to 90 mm Hg, taking antihypertensive medications within 2 weeks before the exam, or self-report of a hypertension diagnosis. Type 2 diabetes was defined as a fasting blood glucose level greater than or equal to 126 mg/dL, receiving glucose-lowering medications, or self-report of a diabetes diagnosis. Renal function was assessed by using estimated glomerular filtration rate (eGFR, mL/min/1.73 m^2^), which was obtained from standard assays.

Television viewing time was categorized as less than 2 hours per day, 2 to 4 hours per day, or more than 4 hours per day. We examined differences in baseline characteristics among categories of television viewing time by using the ANOVA test for continuous variables and the χ^2 ^test for categorical variables. We used multivariable Cox proportional hazards models adjusted for baseline behavioral and health factors (smoking, alcohol use, nutrition, physical activity, education, history of prevalent coronary heart disease, chronic kidney disease, diabetes, and hypertension) and computed hazard ratios (with 95% confidence intervals [CIs]) of each group by using the group reporting less than 2 hours per day of TV viewing as the reference category. A complete case analysis was performed on participants who had information on all covariates. Person-time of follow-up was calculated from the baseline questionnaire data to the date of the event (death) or censoring (loss to follow-up or the end of the study period), whichever occurred first. Data analysis was conducted in 2017.

Covariates in the models were chosen on the basis of a priori knowledge. Model 1 showed the crude association; model 2 adjusted for age; model 3 adjusted for age and sex; and model 4 was the multivariable model adjusted for age, sex, weekly physical activity, nutrition, alcohol use, and smoking. We did not include BMI, diabetes, and hypertension in the model, because these would be in the intermediate pathway from sedentary behavior to mortality. We performed analyses stratified by level of physical activity to determine whether the association between television watching and mortality differed among those who exercised, on the basis of previously described AHA physical activity categories. We also conducted several sensitivity analyses to test the robustness of our results excluding the following: those who died within the first 2 years of the start of the JHS study, those with a BMI less than or equal to 18.5 kg/m^2 ^or greater than or equal to 40 kg/m^2^, participants with an eGFR less than 15 mL/min/1.73 m^2^, and those without a high school diploma. To test the proportional hazards assumption, we tested the interaction between variable and log (person-time), and no violations were found (*P* > .05). We performed all analyses using SAS version 9.4 (SAS Institute, Inc), and used an α level of 0.05 (16).

## Results

The mean age of the 5,289 participants was 55 years, and 64% were women. At baseline, 28.3% of participants watched television less than 2 hours daily, 33.8% watched television for 2 to 4 hours daily and 37.9% watched television 4 hours or more daily (Table 1). The group that watched television more than 4 hours a day (n = 2,002) was slightly older, was less educated, had poorer nutritional status, had a greater percentage of smokers, had higher rates of diabetes, and had higher total cholesterol levels.

**Table 1:** Baseline Characteristics of Participants (N = 5,289), the Jackson Heart Study, 2017^a^

Characteristics	Hours of television Viewing		
	<2 h/d, n = 1,498	2-4 h/d, n = 1,789	≥4 h/d, n = 2,002
**Age, mean (SD), y**	53.8 (12.7)	54.9 (12.7)	57.0 (12.9)
**Women**	65.2	62.1	63.6
**Body mass index, mean (SD), (kg/m2)**	31.0 (6.6)	32.0 (7.4)	32.1 (7.6)
**Education**			
Less than high school diploma	15.2	16.7	27.5
High school diploma or above	84.8	83.3	72.5
**Alcohol consumption (yes)**	46.9	46.4	44.6
**AHA physical activity category**			
Poor health	42.7	46.7	56.6
Intermediate health	35.0	31.9	28.8
Ideal health	22.3	21.4	14.6
**AHA nutrition category**			
Poor health	58.5	60.2	62.9
Intermediate health	40.3	38.8	36.4
Ideal health	1.20	1.06	0.65
**Former or current smoker**	27.4	30.3	38.1
**Hypertension**	51.8	55.6	61.1
**Systolic blood pressure, mean (SD), (mm Hg)**	126.2 (16.9)	127.2 (16.4)	128.7 (17.2)
**Diastolic blood pressure, mean (SD), (mm Hg)**	75.7 (8.4)	75.7 (8.5)	75.7 (9.2)
**Coronary heart disease history**	6.9	6.9	8.6
**Chronic kidney disease history**	4.5	4.6	6.0
**Diabetes mellitus**	18.8	21.2	24.7
**HbA1c, mean (SD)**	5.9 (1.2)	6.0 (1.3)	6.1 (1.4)
**Total cholesterol, mean (SD)**	196.9 (37.7)	198.9 (40.0)	201.5 (41.9)
**HDL cholesterol, mean (SD)**	52.5 (14.5)	51.2 (14.2)	51.8 (15.1)

Abbreviations: AHA, American Heart Association; HbA1c, hemoglobin A1c; HDL, high density lipoprotein.

a Values are shown as column percentages. A total of 5,301 participants completed a baseline clinical examination and questionnaire during 2000–2004. Of these, 12 had missing data on TV watching. Other missing variables are as follows: education, 27; CHD history, 12; CKD history, 31; diabetes status, 73; hypertension, 14; AHA physical activity categorization, 12; AHA nutrition category, 12; smoking status, 18; alcohol status, 47; and occupational status, 13. Data for all variables included in the analysis were collected at the time of exam 1. Mean values are presented for continuous variables unless otherwise indicated.

After a median follow-up of 10 years (interquartile range: 9.0–10.7), 615 deaths occurred. The Figure depicts the cumulative incidence of mortality by television viewing time. In a multivariable Cox regression model adjusting for age, sex, smoking, alcohol use, physical activity, and nutrition, hazard ratios for mortality were 1.48 (95% confidence interval [CI], 1.19–1.83) for television viewing time equal to or greater than 4 hours per day and 1.08 (CI, 0.86–1.37) for television viewing time of 2 to 4 hours per day when compared with those who watched television less than 2 hours per day (*P* trend =.002) (Table 2). When we restricted analyses to those who performed physical activities, the hazard ratios for mortality were 1.45 (95% CI; SD, 1.13–1.86) for television viewing of more than 4 hours per day and 1.10 (95% CI; SD, 0.84–1.45) for 2 to 4 hours per day as compared with the reference category (<2 h/d) (Table 3). In sensitivity analysis the association persisted excluding those who died within the first 2 years, excluding those with a BMI at or below 18.5 or at or above 40 kg/m^2^, eGFR less than 15 mL/min/1.73m^2^, and excluding those without a high school diploma (Table 3).

**Figure:**
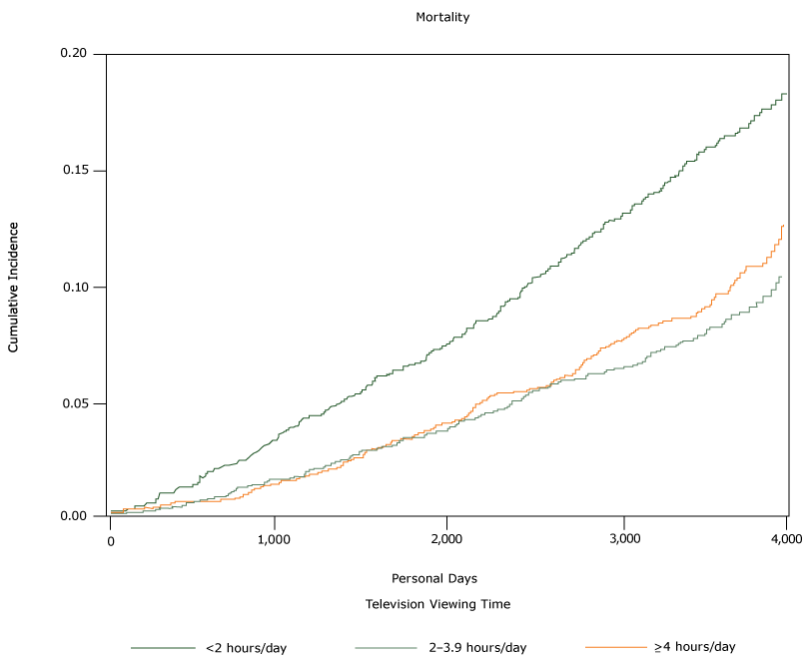
The cumulative incidence of all-cause mortality in relationship to time spent watching television. Data are from 5,289 participants in the Jackson Heart Study (9–13), self-reported in baseline questionnaires administered from 2000 through 2004.

**Table 2:** Analysis of the Association Between Television Viewing Time and All-Cause Mortality, Participants (N = 5,289), the Jackson Heart Study, 2017

Variable	Frequency of Television Watching (h/d)^a^			
	<2 h/d	2-4 h/d	≥4 h/d	*P* Value^b^
Cases/total person-years	126/14,111	174/16,793	315/18,064	NA
Crude incidence/10,000 person-years	89.3	103.6	174.5	NA
Crude model	1 [Reference]	1.16 (0.93–1.46)	1.97 (1.60–2.42)	<.001
Age adjusted model		1.06 (0.84–1.34)	1.61 (1.31–1.98)	<.001
Age and sex adjusted model		1.07 (0.85–1.35)	1.61 (1.31–1.98)	<.001
Multivariable model 1^c^		1.07 (0.85–1.35)	1.50 (1.22–1.85)	<.001
Multivariable model 2^d^		1.08 (0.86–1.37)	1.48 (1.19–1.83)	.002

Abbreviation: NA, not applicable.

a Values are hazard ratio (95% confidence interval) unless otherwise indicated.

b *P* values and hazard ratios were computed using Cox proportional hazard models

c Adjusted for age, sex, physical activity, nutrition, alcohol use and smoking.

d Adjusted for age, sex, physical activity, nutrition, alcohol use, smoking, education and history of prevalent coronary heart disease, chronic kidney disease, diabetes, and hypertension.

**Table 3:** Subgroup Analysis of All-Cause Mortality According to Television Viewing Time, Participants (N = 5,289), the Jackson Heart Study, 2017

Variable	Television Viewing Time, Hazard Ratio (95% Confidence Interval			*P* Value^a^
	<2 h/d	2-4 h/d	≥4 h/d	
**Excluding those who died in the first two years (n = 4,923)**				
Age and sex adjusted model	1 [Reference]	1.12 (0.88–1.42)	1.56 (1.25–1.93)	<.001
Multivariable model^b^		1.12 (0.88–1.44)	1.41 (1.13–1.76)	.005
**Excluding those with BMI ≤ 18.5 (n = 5,123)**				
Age and sex adjusted model	1 [Reference]	1.07 (.85–1.35)	1.59 (1.29–1.96)	<.001
Multivariable model^b^		1.08 (.85–1.37)	1.47 (1.18–1.82)	.003
**Excluding those with BMI ≥ 40 (n = 4,540)**				
Age and sex adjusted model	1 [Reference]	1.11 (.87–1.41)	1.60 (1.28–1.99)	<.001
Multivariable model^b^		1.13 (.88–1.45)	1.47 (1.17–1.85)	.001
**Excluding those with eGFR < 15 (n = 5,103)**				
Age and sex adjusted model	1 [Reference]	1.10 (.86–1.39)	1.68 (1.35–2.08)	<.001
Multivariable model^b^		1.09 (.86–1.39)	1.52 (1.23–1.89)	<.001
**Excluding those with low leisure physical activity (n = 3,519)**				
Age and sex adjusted model	1 [Reference]	1.08 (.83–1.41)	1.51 (1.19–1.92)	.004
Multivariable model^b^		1.10 (.84–1.45)	1.45 (1.13–1.86	.004
**Excluding those without a high school diploma (n = 3,175)**				
Age and sex adjusted model	1 [Reference]	1.09 (.77–1.54)	1.79 (1.30–2.47)	.001
Multivariable model^b^		1.01 (.71–1.45)	1.58 (1.13–2.19)	.003

Abbreviations: AHA, American Heart Association; BMI, body mass index (kg/m^2^); eGFR, estimated glomerular filtration rate.

a *P* values and hazard ratios were computed using Cox proportional hazard models.

b The multivariable model was adjusted for age, sex, nutrition, alcohol use, and smoking. We excluded participants who had none to low levels of physical activity according to the American Heart Association physical activity categories (15).

## Discussion

To our knowledge, this is the first study to describe the association between television viewing time and all-cause mortality among African American men and women in the continental United States. Our study suggests that television-viewing time is associated with mortality even among those who are obese or who perform regular leisure-time physical activity. Prior studies conducted in various populations found a similar association between television viewing time and mortality (17–20). In the Australian Diabetes, Obesity, and Lifestyle Study, which included 8,800 Australian adults aged 25 years and older, the hazard ratio for mortality for each 1 hour increment in television viewing time per day was 1.11 (95% CI; SD, 1.03–1.20) after 6.6 years of follow-up (17). The Multiethnic Cohort Study consisted of men and women from Hawaii and Los Angeles aged 45 to 75 years from 5 racial/ethnic groups (18). At a follow-up time of 13.7 years, the hazard ratio for all-cause mortality for African Americans (n = 7,252) who sat (including television viewing) for more than 5 hours per day compared with those who sat for less than 1 hour per day was 1.37 (SD, 1.14–1.66). This was after adjusting for age, education, race/ethnicity, smoking, alcohol consumption, energy intake, physical activity, and history of hypertension or diabetes. Although the effect size in our study was in the same direction as that in the Multiethnic Cohort Study, the hazard ratio in our population was higher, perhaps because of differing lifestyle factors. For instance, a substantial portion of the JHS participants consumed a southern diet, which typically consists of high amounts of sugars and saturated fats, and they may have had a more sedentary lifestyle. Also, a significant percentage of JHS participants had hypertension, which is a predisposing risk factor for cardiovascular events and thus, all-cause mortality. Similarly, the EPIC (European Prospective Investigation of Cancer)–Norfolk study, which included 25,633 residents of Norfolk, England, aged 45 to 79 years, reported a hazard ratio of mortality of 1.04 (95% CI; SD 1.01–1.09) for each 1-hour increase in television viewing time after a mean follow-up of 9.5 years (19). After 6.6 years of follow-up, the National Institutes of Health–American Association of Retired People Diet and Health Study, a prospective cohort of adults aged 50 to 71, found a 28% greater mortality risk (95% CI; SD 1.21–1.34) for those who watched television more than 5 hours per day compared with those who watched for less than 3 hours daily (20).

Contrary to these findings, a study conducted among 7,350 adults aged 20 years or older who participated in the National Health and Nutrition Examination Survey from 1999–2002, did not find a significant association between mortality and television viewing or video game or computer screen time (HR: 1.30; 95% CI; 0.82–2.05) after approximately 5 years of follow-up and after adjusting for age, sociodemographic and behavioral factors, health status, insurance coverage, and prevalent chronic conditions (21).

Several biological and behavioral mechanisms are postulated that may explain how television viewing time, as a marker of sedentary time, may affect mortality. Television viewing may be associated with increased consumption of snack foods, leading to increased total calorie intake (22). Also, it may substitute for leisure activity time and thus lead to reduced energy expenditure. This would then predispose to the development of obesity, metabolic syndrome, and cardiovascular disease. Cohort studies have found that sedentary time is linked to blood glucose and lipid levels (23). Preclinical studies have shown that sedentary time is associated with lipoprotein lipase activity, a protein involved in plasma triglyceride catabolism, HDL cholesterol, and other metabolic risk factors (24). A study on vascular aging found that increasing television viewing time directly correlated with an increase in peripheral augmentation index, a marker of arterial stiffness (25).

Our study had limitations. Television viewing time was self-reported in questionnaires. This could have led to some misclassification because of social desirability bias. Furthermore, it is assumed that television viewing time is part of sitting time, thus constituting sedentary behavior. Physical activity has been validated with accelerometer and pedometer data in JHS. Higher physical activity was associated with higher raw accelerometer and pedometer counts with Spearman correlations of ρ = 0.24 and ρ = 0.32, respectively (26). Some people may report what they perceive is socially acceptable for daily television viewing time rather than their true television viewing time. As with other observational studies, confounding resulting from unmeasured or unaccounted for variables cannot be excluded. It is plausible that those who spend less time watching television may also have other healthy behaviors that may be part of a group of behaviors inversely associated with mortality. Conversely, those who spend more time watching television may be less likely to engage in regular moderate-to-intense physical activity or may be more likely to eat a less healthy diet, making them more prone to disease (18). Reverse causation is a concern, because those with chronic or debilitating health conditions may spend more time sitting. However, we adjusted for participants with baseline chronic conditions such as diabetes, hypertension, coronary heart disease, and chronic kidney disease. We also conducted several subgroup analyses such as excluding those who died within the first 2 years of follow-up after baseline questionnaires, which did not change the findings. Given that our population consists of African Americans from Jackson, Mississippi, who may have different dietary patterns and physical activity behaviors than African Americans from other regions; the results may not be entirely generalizable to others. However, the physiologic mechanisms of sedentary behavior and its impact on mortality are not likely to differ across populations.

Our study also has several strengths. The JHS study is one of the largest studies of heart health among African Americans in the United States. Additional strengths include a prospective design, adequate follow-up duration, a standardized ascertainment of the exposure question and outcomes, and incorporation of detailed lifestyle and behavioral factors in the analysis.

Our findings suggest that greater television viewing time is associated with a higher risk of all-cause mortality among African Americans, even after adjustment for leisure-time physical activities, and may serve as another measure of sedentary lifestyle for potential public health interventions. Such interventions should promote reducing sedentary time in addition to advocating for increased physical activity among African Americans.
